# Polyvinylidene Fluoride Membrane Via Vapour Induced Phase Separation for Oil/Water Emulsion Filtration

**DOI:** 10.3390/polym13030427

**Published:** 2021-01-29

**Authors:** Normi Izati Mat Nawi, Nur Rifqah Sait, Muhammad Roil Bilad, Norazanita Shamsuddin, Juhana Jaafar, Nik Abdul Hadi Nordin, Thanitporn Narkkun, Kajornsak Faungnawakij, Dzeti Farhah Mohshim

**Affiliations:** 1 Chemical Engineering Department, Universiti Teknologi PETRONAS, Seri Iskandar 32610, Perak, Malaysia; normi_16000457@utp.edu.my (N.I.M.N.); rifqahsait@gmail.com (N.R.S.); nahadi.sapiaa@utp.edu.my (N.A.H.N.); 2 HICoE-Centre for Biofuel and Biochemical Research, Institute of Self-Sustainable Building, Universiti Teknologi PETRONAS, Seri Iskandar 32610, Perak, Malaysia; 3 Faculty of Integrated Technologies, Universiti Brunei Darussalam, Jalan Tungku Link BE1410, Brunei; norazanita.shamsudin@ubd.edu.bn; 4 Advanced Membrane Technology Research Center (AMTEC), School of Chemical and Energy Engineering (SCEE), Universiti Teknologi Malaysia (UTM), Johor 81310, Malaysia; juhana@petroleum.utm.my; 5 National Nanotechnology Center (NANOTEC), National Science and Technology Development Agency (NSTDA), 111 Thailand Science Park, Pathum Thani 12120, Thailand; thanitporn.nar@ncr.nstda.or.th (T.N.); kajornsak@nanotec.or.th (K.F.); 6 Petroleum Engineering Department, Universiti Teknologi PETRONAS, Seri Iskandar 32610, Perak, Malaysia; dzetifarhah.mohshim@utp.edu.my

**Keywords:** membrane fouling, VIPS, PVDF membrane, oil/water emulsion, oily wastewater, membrane technology, antifouling

## Abstract

Membrane-based technology is an attractive option for the treatment of oily wastewater because of its high oil removal efficiency, small footprint and operational simplicity. However, filtration performance is highly restricted by membrane fouling, especially when treating oil/water emulsion as a result of strong interaction between oil droplets and the hydrophobic property of the membrane. This study explores the fabrication of polyvinylidene fluoride (PVDF)-based membrane via the vapour induced phase separation (VIPS) method while incorporating polyvinyl pyrrolidone (PVP) as a hydrophilic additive to encounter membrane fouling issues and improve membrane filterability. The resulting membranes were characterized and tested for oil/water emulsion filtration to evaluate their hydraulic, rejection and anti-fouling properties. Results show that the changes in membrane morphology and structure from typical macrovoids with finger-like substructure to cellular structure and larger membrane pore size were observed by the prolonged exposure time from 0 to 30 min through the VIPS method. The enhanced clean water permeability is attributed to the addition of PVP–LiCl in the dope solution that enlarges the mean flow pore size from 0.210 ± 0.1 to 7.709 ± 3.5 µm. The best performing membrane was the VIPS membrane with an exposure time of 5 min (M-5), showing oil/water emulsion permeability of 187 Lm^−2^ h^−1^ bar^−1^ and oil rejection of 91.3% as well as an elevation of 84% of clean water permeability compared to pristine PVDF developed using a typical non-solvent induced phase separation (NIPS) method. Despite the relatively high total fouling, M-5 was able to maintain its high permeability by water flushing as a simple operation for membrane fouling control. The performance was achieved thanks to combination of the large mean flow pore size and hydrophilic property from residual PVP in the membarne matrix. Overall, the results demonstrate the potential of the optimum VIPS method in the presence of PVP and LiCl additives for oil/water emulsion treatment.

## 1. Introduction

Improper discharge of wastewater leads to environmental and ecological pollution. It is promoted by massive industrial activities including oil and oleochemicals. Rapid industrial development in the oil and gas, petrochemical, pharmaceutical and food industries have led to large production of oily wastewater [1]. For instance, production of the palm oil mill effluent (POME) is one of main industrial causes of the production of oily wastewater in Southeast Asian countries such as Indonesia, Malaysia and Thailand, which produced approximately 36, 21 and 2 million × 10^3^ kg of palm oil, respectively, in 2017 [2,3,4]. These values are estimated to be increasing annually, which eventually results in increasing discharge of oily wastewater [2]. It is worth mentioning that oil/water emulsion has been acknowledged as one of the most persistent liquid waste sources polluting the environment, as it may affect the resources of drinking water and groundwater, endanger human health as well as aquatic life and affect the production of crops [5,6,7]. Hence, it is crucial to properly treat the wastewater that contains oil/water emulsion to lower the oil content for either reuse or discharge purposes. 

Various technologies have been employed for treatment of the oil/water emulsion. They include conventional physical and chemical methods such as sand filter, cyclones, adsorption, photocatalytic treatment, and oxidation, as well as electro-chemical processes [1]. Unfortunately, most of them suffer from a few limitations such as high investment costs, large space requirements for installation and less effectiveness in treating emulsion with oil droplet sizes of less than 2 μm [1,5]. Thus, the membrane-based process has emerged as an attractive option for treating oil/water emulsion [1]. Despite its advantages, such as high separation efficiency, less energy usage, smaller footprint, etc., membrane separation often experiences membrane fouling due to strong interaction between oil droplets and the membrane surface, and unfavorable pore structure and morphology [8,9,10,11,12]. 

Studies reported that plain polymeric membranes such as polyvinylidene fluoride (PVDF) pose hydrophobic properties and thus increase the membrane fouling propensity, which deteriorates the hydraulic performance [10,13]. Membrane fouling also leads to a severe decline in flux as well as impeding long-term operation of a membrane process [14]. Thence, it is vital to custom make the membrane material so that it is tailored to specific applications in order to increase the sustainable flux and, at the same time, limit the membrane fouling [15]. For instance, Fahrina et al. [16] incorporated bio-based ginger extract as additive to enhance biofouling resistance of PVDF membrane. The results show that the addition of ginger extract enhances the membrane surface hydrophilicity, which elevates the pure water flux by up to 62%. Meanwhile, Zuo et al. [17] modified the PVDF membrane by introducing polydopamine as the additive by using the thermal induced phase separation (TIPS) method with cold air as a quenching bath. The optimal membrane sample shows permeability of 2600 Lm^−2^ h^−1^ bar^−1^ for oil/water emulsion filtration with >98% of oil rejection. Recently, Chiao et al. [18] transformed PVDF ultrafiltration membrane to a negatively charged loose nanofiltration membrane through UV-grafting of acrylic acid for dye removal from textile wastewater. It was found that the optimal exposure time of PVDF membrane to UV light is 5 min. The membrane showed high dye recovery of 99% and low salt rejection of less than 15% along with pure water flux of 26 Lm^−2^ h^−1^ bar^−1^. 

Membrane development via the incorporation of hydrophilic additives such as polyvinyl pyrrolidone (PVP) can indeed obviate the adhesion of oil droplets on the membrane surface, as reported by Padaki et al. [1]. However, simply blending the hydrophilic additives into casting solution by employing the non-solvent induced phase separation (NIPS) for membrane fabrication results in leaching out of the additive during membrane fabrication and relatively small pore size [19]. Since the oil droplet sizes in oil/water emulsion are relatively large, application of microfiltration membrane is attractive to boost the flux. The NIPS–PVDF-based membranes are generally limited to the production of microfiltration membrane when incorporating additives. The properties of the resulting membranes are strongly affected by the trade-off of kinetic and thermodynamic effects during the demixing process.

In 1918, the vapour induced phase separation (VIPS) method was introduced by Zsigmondy and Bachmann to induce phase separation, which occurs when there is penetration of the nonsolvent from a vapour phase to a polymer solution, thus inducing phase inversion [20]. In contrast to NIPS, the nonsolvent phase in VIPS is a gas and the nonvolatile nonsolvent is originally contained in the volatile solution. This results in the non-solvent being enriched in the casting solution during the process of a controlled solvent evaporation. The VIPS method also enables better controlling of the desired membrane morphology during the separation of phases, which can be attained by proper monitoring and regulating of the phase inversion process during the fabrication step [20]. Generally, there are four main morphologies that are frequently obtained using the VIPS process, namely symmetric cellular [21], asymmetric cellular [22], symmetric nodular [23,24] and symmetric bi-continuous structures [25]. Tsai et al. [26] proved that the morphologies of the developed polysulfone (PSF)-based hollow fiber membranes were strongly affected by air-gap length and ambient humidity. It was found that the air-gap length required for the suppression and re-suppression of macrovoids reduced with elevated relative humidity. Meanwhile, a recent study by Dehban et al. [27] suggests that increasing VIPS time would enhance the membrane pore size and pure water flux by up to 55% when the VIPS time was extended from 0 to 30s. 

Considering the advantages of VIPS process, this study explores fabrication of PVDF-based membrane via this method to address the membrane fouling problem in oil/water emulsion filtration, as well as to incorporate hydrophilic additives such as PVP and lithium chloride (LiCl). The VIPS method is expected to provide additional degrees of freedom in tuning the surface properties of the resulting membrane. Membrane characterizations were delicately performed to determine the effect of fabrication parameters with respect to exposure time on the membrane’s properties and their hydraulic performance in treating oil/water emulsion as well as their antifouling properties. 

## 2. Materials and Methods 

### 2.1. Membrane Fabrication

**Figure 1:**
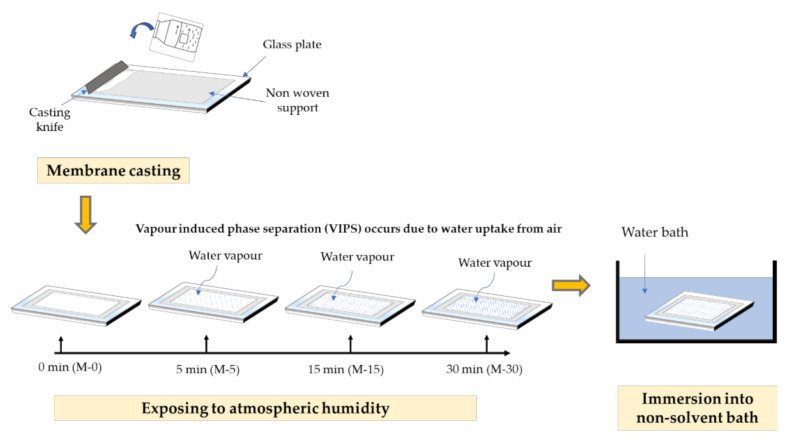
Illustration of membrane preparation using the vapour induced phase separation VIPS method.

The membranes were fabricated using PVDF (average M_w_ ~534,000 by gel permeation chromatography (GPC), Sigma-Aldrich, St Louis, MO, USA), dimethylacetamide DMAC (Sigma-Aldrich, St Louis, MO, USA) and deionized water as the polymer, solvent and non-solvent, respectively. A pristine PVDF membrane with 15 wt% PVDF was fabricated as a benchmark for this study. A concentration of 3 wt% of PVP (average M_w_ ~10,000, Sigma-Aldrich, St Louis, MO, USA) was incorporated as the hydrophilic additive and a small amount of LiCl (0.1 wt%) (AcrosOrganics, Fair Lawn, NJ, USA) was added as pore former. All of the chemicals were mixed in a schott bottle and stirred at 60 °C for at least 24 h or until it homogeneously mixed. Before casting, the dope solution was left idle overnight to eliminate air bubbles in order to avoid membrane defects. Then, the solution was casted with a wet thickness of 200 µm on a non-woven support by using a casting knife. The cast polymer film was exposed to atmospheric humidity for varied durations of 5, 15 and 30 min before being immersed in the coagulation bath containing water which acts as the non-solvent. The cast film was left in the bath for 24 h to completely remove the solvent trace from the membrane matrix. Figure 1 illustrates the step by step process of membrane fabrication using the VIPS method, while Table 1 shows a summary of the membrane fabrication parameters. 

### 2.2. Membrane Characterization

The properties of the developed membranes were evaluated in terms of membrane morphology, pore size distribution, hydrophilicity, chemical surface distribution and clean water permeability. The images of the membranes’ surfaces and cross-sections were determined using scanning electron microscopy (SEM, FEI Quanta-250, Thermo Fisher Scientific, Waltham, MA, USA). Prior to testing, the samples were immersed in liquid nitrogen to freeze-fracture the samples and then were coated with a thin gold layer. The pore size distribution of the resulting membranes was determined using a capillary flow porometer by Porolux 1000 (Berlin, Germany). The membranes’ surface hydrophilicity were measured using a goniometer (Ramé-Hart 260, Succasunna, NJ, USA) to obtain the contact angle values, while fourier transform infrared spectroscopy (FTIR) (PerkinELmer, Inc., Waltham, MA, USA) withspectra ranging from 400 to 4000 cm^−1^ and an X-ray photoelectron spectrometer (XPS, K-Alpha^TM^, Thermo Scientific, Walthan, MA, USA) were used to determine the chemical composition on the membranes’ surfaces. 

### 2.3. Filtration Configuration

**Figure 2:**
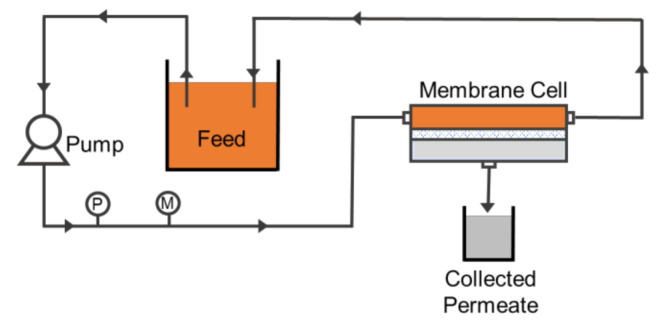
Laboratory setup illustration of the filtration system.

The hydraulic performance of the developed membranes was evaluated by using a cross-flow microfiltration, as illustrated in Figure 2. The filtration was operated at a fixed pressure difference of 0.2 bar with a fixed effective membrane area of 0.0036 m^2^. The filtration was started by filtering clean water for compaction purposes and for measuring the clean water permeability (CWP). Both compaction and CWP filtration were conducted for around 90 min, in which the first 60 min involved compaction, followed by the CWP measurement. After completing the CWP filtration, 5 cycles of oil/water emulsions filtration were subsequently conducted. The oil/water emulsion filtration was performed for 30 min, followed by flushing with clean water for 5 min as membrane cleaning, which was considered as one cycle. The feed preparation of oil/water emulsion was detailed elsewhere [24]. The filtrations were then continued for another four cycles. A small volume of feed samples was later analysed using a particle size and zeta potential analyzer (Zetasizer Nano ZSP, Malvern, PA, USA) to map the oil droplet size distribution.

The flux (*J*), permeability (*L*) and oil rejection (*R*) were calculated using Equations (1)–(3), respectively:

(1) $$J=\;\frac{∆V}{A\;∆t}$$

(2) $$L=\;\frac{∆V}{A\;∆t\;∆p}$$

(3) $$R\;=\;\frac{C_{feed}-\;C_{permeate}}{C_{feed}}\times \;100\;$$

where ∆*V* is the change in volume of draw solution (L), *A* the effective area of the membrane (m^2^), ∆*t* is the time taken (h), ∆*P* is the pressure difference (bar), *C_feed_* is the concentration of feed (ppm) and *C_permeate_* is the concentration of permeate (ppm). 

### 2.4. Anti Fouling Analysis

For the anti-fouling analysis, total fouling (*T_F_*), reversible fouling (*R_F_*) and irreversible fouling (*IR_F_*) were evaluated by using Equations (4)–(6), respectively:

(4) $$T_{F}=\;\frac{L_{CW}-L_{O/W}}{L_{CW}}\;\times 100\%$$

(5) $$R_{F}=\;\frac{L_{W}-\;L_{O/W}}{L_{CW}}\;\times 100\%$$

$$IR_{F}=\;T_{F}-R_{F}$$

(6) $$R_{F}=\;\frac{L_{W}-\;L_{O/W}}{L_{CW}}\;\times 100\%$$

where *L*_CW_ is the permeance of clean water (Lm^−2^ h^−1^ bar^−1^), *L*_O/W_ is the permeance of oil/water (*L* m^−2^ h^−1^ bar^−1^) and *L*_W_ is the permeance of water (Lm^−2^ h^−1^ bar^−1^).

## 3. Results and Discussion

### 3.1. Membrane Characterization

#### 3.1.1. Membrane Morphology

**Figure 3:**
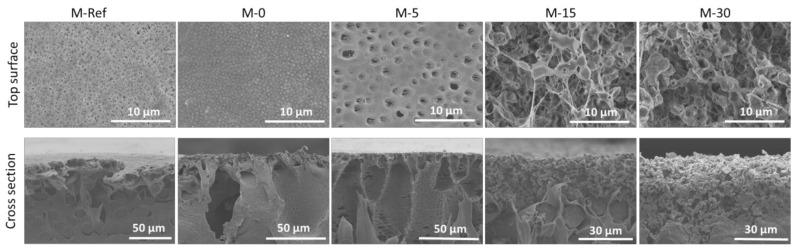
Surface and cross-sectional morphology of the membranes.

Figure 3 shows the morphological images of the plain PVDF membrane (M-Ref) as well as the modified PVDF membranes (M-0, M-5, M-15 and M-30). The results demonstrate a clear relationship between the exposure time with the morphology of resulting PVDF membranes. The pristine PVDF (M-Ref) and M-0 membranes both have typical asymmetric structures with dense top layers and have large macrovoids with finger-like substructures near the top surface. According to He et al. [28], the finger-like structure is typically formed through pore initiation and growth. During the phase inversion, there is development of polymer-rich as well as polymer-lean phases during liquid–liquid demixing through the nucleation and growth mechanism. The nuclei of the polymer-lean phases form a finger-like structure followed by the formation of more nuclei beneath the top layer. Moreover, the state of the solution in the border of the phase separation usually influences the growth of the nuclei [28]. Hence, the formation of large macrovoids with finger-like substructures can be explained through the process of instantaneous demixing in which the polymer precipitates while formation of a solid occurs rapidly after immersion in the non-solvent bath [29]. Polymer precipitation happens due to the low miscibility between the PVDF polymer and water (non-solvent). In addition, due to the miscibility between the non-solvent (water) and the solvent (DMAC), diffusional flow of the solvent and non-solvent, at various points of the film’s top layer and sublayer, has occurred. This occurrence indeed causes the formation of nuclei of a polymer-poor phase [30]. 

Figure 3 also shows that the macrovoids formed in M-0 are smaller as compared to M-Ref. This phenomenon portrays that the macromolecular nature of the additive (PVP) is responsible for the suppression of macrovoids’ formation [31]. At the same time, the diffusion between both polymers often should be slower as compared to the diffusion of the solvent and non-solvent in the casting solution. On the other hand, the addition of a hydrophilic additive, such as PVP, to the casting solution can also enhance the thermodynamic instability of the polymer solution, which eases the formation of macrovoids, thus leading to instantaneous demixing [30]. Judging from the result in Figure 3, the role of PVP in enhancing the viscosity of the dope solution is more pronounced, resulting in the suppression of the macrovoids. 

As illustrated by the SEM images of the top surface morphology in Figure 3, M-0 shows the formation of a porous layer with a granular structure on the membrane surface. The SEM images also demonstrate that the top surface layer of the membranes became more porous as the time gap was extended. The morphologies of the VIPS membranes portray drastic changes from asymmetric to symmetric as the exposure time was extended. In the VIPS process, the non-solvent vapour present in the humid air gradually interacted with the dope solution during the exposure time before being immersed in the water bath [32]. It is worth mentioning that vapour often slowly penetrates into the wet film as the wet film is exposed to the water vapour, where the phase separation is induced through formation of the polymer-rich and polymer-lean phase on the top of the cast film.

Extended exposure time leads to the coarsening process of the polymer-lean phase. The polymer-lean phase eventually produces pores, as these generate the cellular structure justified from the surface SEM image of M-5 in Figure 3, as also reported by others [33]. Hence, the available time is limited for the growth of nuclei at shorter exposure times, which results in smaller macrovoids. However, in terms of mechanical properties, membranes at shorter exposure times are expected to have enhanced mechanical properties as the structures are more interconnected. Furthermore, as claimed by other studies [29,32], the formation of morphology transforms from finger-like macrovoids to sponge-like pores, as is commonly found for VIPS-based membrane. Unfortunately, based on SEM images for both M-15 and M-30 membranes, in terms of morphology, both of them are not well intact. Meanwhile, in terms of mechanical properties, M-30 is the weakest as the polymer clusters are loosely attached to each other.

#### 3.1.2. Membrane Pore Size and Distribution

**Figure 4:**
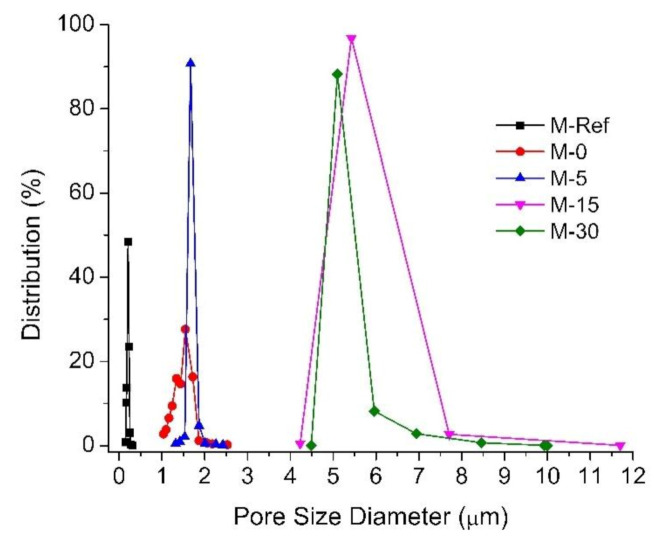
Pore size distribution of the resulting membranes.

Figure 4 shows the pore size distribution of the developed membranes. The addition of PVP and LiCl into the dope solution resulted in a slight increase in the membrane mean pore size from 0.210 ± 0.1 µm (M-Ref) to 1.549 ± 0.6 µm (M-0). The range of the pore size of the VIPS membrane is attractive to separate oil/water emulsion droplets. Application of the VIPS method obviously improves the mean pore size. Extending the exposure time before the process of immersion would remarkably enlarge the pore size of 1.549 ± 0.6, 1.67 ± 0.5, 7.709 ± 3.5 and 6.942 ± 2.5 µm for M-5, M-15 and M-30, respectively. Indeed, this justifies the morphological images of the resulting modified membranes, as shown in Figure 3, where the addition of PVP and LiCl as additives influences the structure of the membranes.

The obvious difference in pore size between the plain PVDF membrane (M-Ref) and the rest (M-0, M-5, M-15 and M-30) can be explained due to the addition of PVP and LiCl as additives, which creates a thermodynamic rather than kinetic impact. The presence of PVP increases the viscosity, thus impeding the phase-separation kinetics but significantly enhancing the thermodynamics for the phase separation due to its good hydrophilicity [29]. Furthermore, the increasing trend of the membrane pore size can be explained by the effect of slow non-solvent penetration processes due to the phenomenon of solid–liquid demixing during the exposure to humid air through the VIPS. As reported by Marino et al. [32], in the VIPS process, nucleation plays a crucial role in the polymer-lean phase as well as contributing to the formation of highly porous membrane. Therefore, it is recognized that through the polymer chains, an increase in solution viscosity eventually limits water passage, as well as the formation of spongy matrix and a more porous top surface, thus leading to the formation of pores of a larger mean size. 

#### 3.1.3. Surface Contact Angle

**Figure 5:**
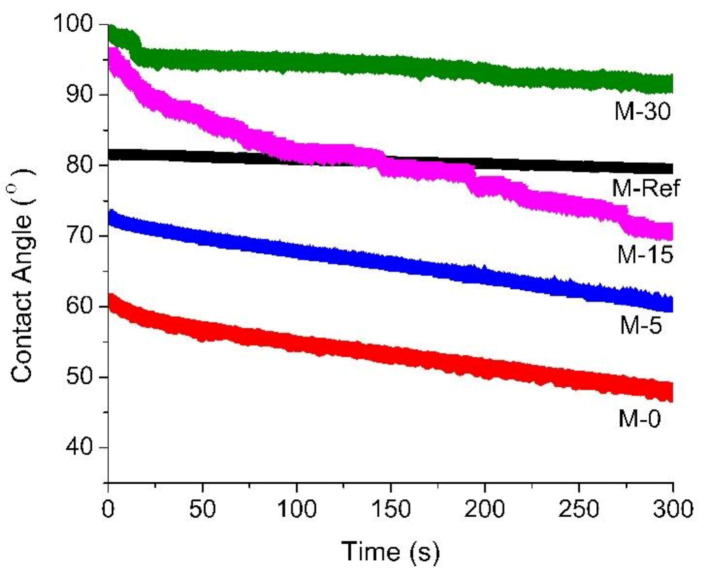
Dynamic contact angle of the developed membranes.

Figure 5 portrays the dynamic water contact angle of the resulting membrane samples. The surface contact angle is used to evaluate the surface hydrophilicty of the membrane. Hence, higher contact angle specifies the hydrophobic nature of the material. It is clearly observed that the addition of PVP as an additive into the polymer solution significantly influences the membrane morphology by affecting the pore formation as well as the hydrophilicity of the membrane. 

The contact angle of M-Ref is much higher than M-0, implying that M-Ref possesses greater hydrophobic properties. M-30 shows the highest contact angle, most likely because of its double-roughness structure consisting of the mixture of macrostructure and microstructure, which is consequently attributed to its improved hydrophobic properties. It is believed that this condition occurs for M-30 not because of the PVP wash out, but because the extended exposure time indeed causes the membrane surface to be rougher, explaining the higher value of the contact angle. 

M-0 exhibits the lowest contact angle value which indicates that it has greatest hydrophilic properties as compared to other modified PVDF membranes, then followed by M-5 and M-15. In addition, M-15 initially portrays the same phenomenon as M-30 which is the *lotus effect*, but then M-15 eventually shows the property of hydrophilicity as the contact angle decreases over the observed time. Furthermore, hydrophobicity properties of the membrane surface contributed to the surface roughness (consisting of both macrostucture and microstructure), which was overwhelmed by the presence of PVP that resides on the membrane surface, thus reducing the contact angle value as a function of time. Both M-0 and M-5 show flat surface morphology with relatively low pore size, which indicates their ability to retain the PVP to impose hydrophilicity. 

#### 3.1.4. Fourier Transform Infrared (FTIR)

**Figure 6:**
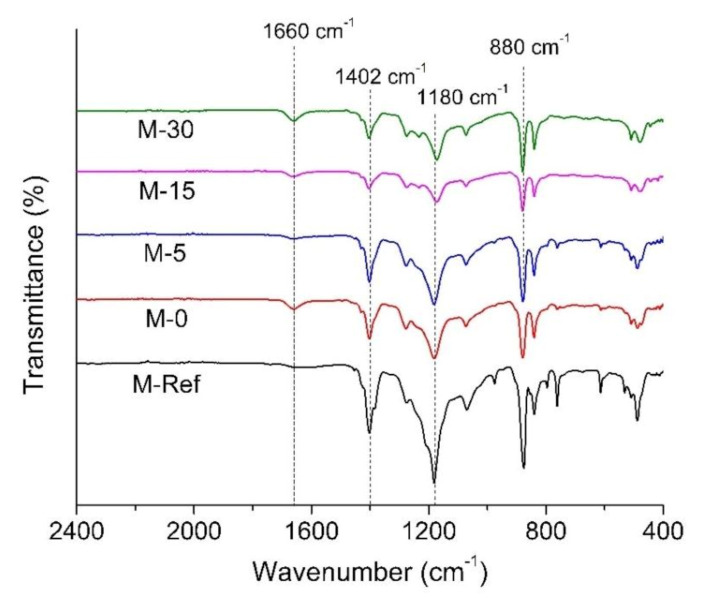
FTIR spectra of the resulting membranes.

The FTIR spectra in Figure 6 implies that VIPS can positively influence the membrane surface hydrophilicity. The results reveal that all of the developed membranes portray peaks at 1402 cm^−1^ and 1180 cm^−1^, indicating the C–H bending vibration and C–F stretching vibrations, respectively, from the PVDF chain. The strong peak at a wavenumber of 880 cm^−1^ also represents the CF_2_ symmetric stretching. 

Furthermore, it is also interesting to find that there is new peak that can be observed at a wavenumber of 1660 cm^−1^ for all modified PVDF membranes, which might be attributed to the presence of residual PVP. As reported by Masuelli et al. [34], the band of carbonyl in the pyrrolidone group often occurs at a wavenumber of 1688 cm^−1^ which indicates the presence of PVP. Consequently, as claimed by Han et al. [35], the characteristic peaks of C=O bond in PVP are observed at 1660 cm^−1^ which represent the presence of the carbonyl group in PVP. It is worth mentioning that during process of phase inversion, hydrophilic additives such as PVP could leach out from the cast film to the nonsolvent [36,37]. Thus, it can be discovered that the prolonged exposure time in the VIPS process allows the formation of semi-solid form on the cast film in order to prevent the leaching of PVP from the matrix of the polymer. However, according to the relative peak intensity around 1660 cm^−1^, the most intense peak is shown for M-0 followed by M-5, M-15 and M-30, suggesting that high exposure time promotes additive leaching. The leaching of additive can also be justified by the larger pore size of the membrane prepared under longer exposure times.

#### 3.1.5. Surface Chemical Composition

By analysis through energy dispersive X-ray (EDX) mapping, the distribution of elemental composition in the membrane sample has been summarized in Table 2. This analysis was performed in order to confirm the incorporation of the PVP in the PVDF-based membrane matrix. The results suggest the presence of Carbon (C), Fluoride (F), Oxygen (O) as well as Nitrogen (N) in each of the membrane samples. In comparison to M-Ref, increasing percentages of composition of oxygen elements are observed on the modified PVDF membranes. The increase in the oxygen element confirms the presence of PVP in the PVDF membrane matrix, promoted by small pore size for M-0 and the formation of immobile film on top of the cast film during exposure to humid air for the VIPS membranes.

The presence of the N element also indicates the presence of PVP in the membrane matrix. The N element originated from the amide carbonyl group [34]. In general, the simple functional group of amide carbonyl consists of two hydrogen atoms which are bonded to Nitrogen (–CONH_2_), hence explaining the presence of component N in M-15 as well as M-30, as shown in Table 2. 

**Figure 7:**
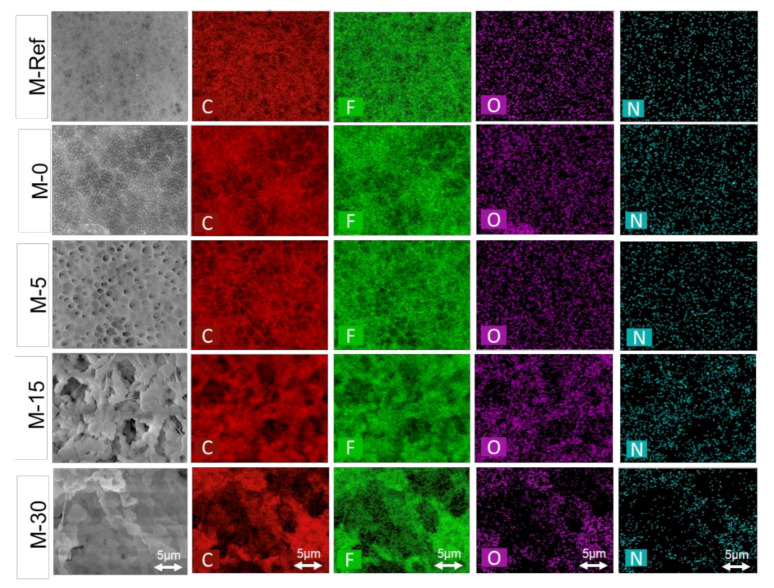
EDX mapping for main elements (C, F, O, and N) present in the resulting membranes.

Figure 7 illustrates the EDX mapping for the elements present in the membrane samples. This EDX analysis was conducted to study the elemental distribution on the membrane surface. Since the mapping was obtained from SEM images, the structure of the membrane surface remarkably affects the elements’ distribution. The flat surface without large pores of M-Ref and M-0 caused all elements to be well dispersed compared to other membranes with relatively large voids (M-15 and M-30).

**Figure 8:**
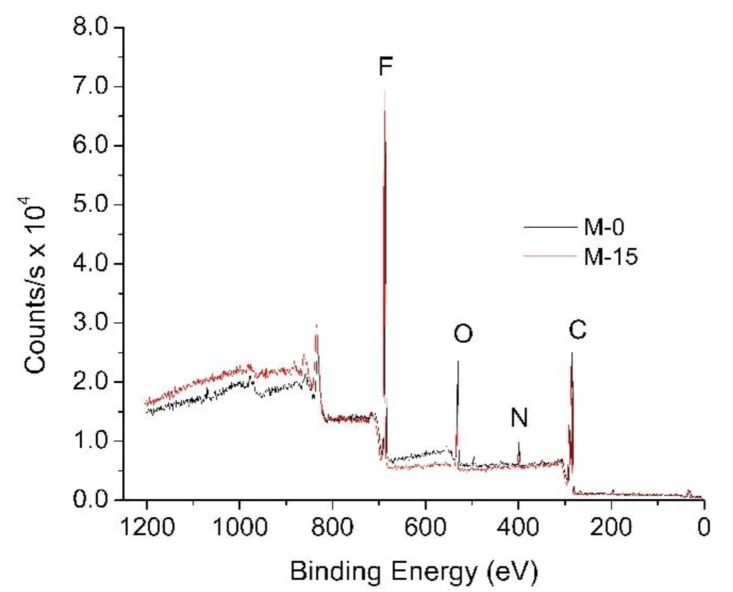
XPS wide scan spectra of M-0 and M-15 membranes.

XPS analysis was conducted to quantify the elemental compositions of the modified PVDF membranes specifically for M-0 and M-15. This characterization aimed to further confirm the effect of extension time in enhancing the residual of PVP in the PVDF membrane matrix. The dope solution as well as the nonsolvent for both membranes are similar. The only things that differ in both of these membranes are the exposure times. As shown in Figure 8, M-0 has a higher oxygen peak as compared to M-15. It suggests that M-0 retained more PVP in the membrane matrix, supporting the findings of FTIR analysis. The higher retention of PVP can be explained by the smaller mean flow pore size of the membrane. The higher retention of PVP in M-0 also leads to a lower contact angle, hence more hydrophilic properties. 

#### 3.1.6. Clean Water Permeability

**Figure 9:**
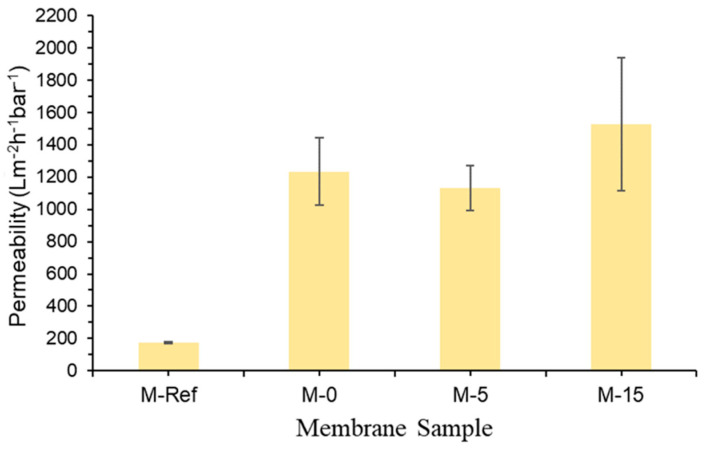
Clean water permeability of the developed membrane samples.

Figure 9 demonstrates the clean water permeability of all membrane samples, depicting the clear advantage of the VIPS method in reducing the intrinsic membrane resistance. The clean water permeability of M-30 is not included because the membrane was physically damaged due to its poor mechanical structure. It was also excluded for the oil/water emulsion filtration.

Generally, the findings prove that the morphology as well as the pore size of the membrane influence its permeability, as also reported by Marino et al. [32]. The M-Ref has the lowest value of clean water permeability because it has the smallest mean flow pore size. The small mean pore size restricts the transport of water droplets across the membrane film.

The results also suggest that M-15 has the highest permeability of 1527.78 ± 411.11 Lm^−2^ h^−1^ bar^−1^ followed by M-0 (1233.33 ± 147.22 Lm^−2^ h^−1^ bar^−1^), M-5 (1130.56 ± 137.50 Lm^−2^ h^−1^ bar^−1^) and lastly M-Ref (179.17 ± 4.17 Lm^−2^ h^−1^ bar^−1^). It is believed that the highest permeability shown by M-15 is due to it having the largest pore size and good hydrophilicity. Even though the mean flow pore size of M-5 is slightly larger than M-0, the latter shows higher hydrophilicity, which indicates that hydrophilicity is more sensitive than the mean flow pore size towards permeability. 

### 3.2. Effect of Exposure Time on Membrane Hydraulic Performance

#### 3.2.1. Permeance Recovery Analysis

**Figure 10:**
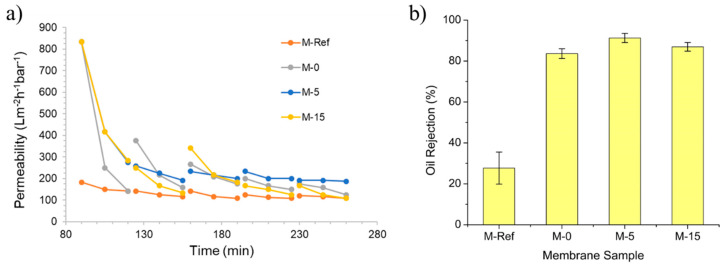
The evolution of permeability over time during oil/water emulsion filtration (**a**) and its permeate quality in terms of oil rejection (**b**) of the developed membranes.

Figure 10 exhibits the evolution of permeability over time for oil/water emulsion filtration and its oil rejection. The first part of the filtration was performed for 90 min by using clean water as the feed for membrane compaction. Subsequently, the second part was conducted by using oil/water emulsion as the feed for 30 min, followed by flushing with clean water for 5 min in order to complete one filtration cycle. The oil/water emulsion filtration was conducted in five cycles. The permeabilities of clean water and oil/water emulsion in every part were recorded to evaluate the trend. The depreciation of the permeability over time indicates the occurrence of membrane fouling. It is nearly impossible to have zero-fouling in any pressure-driven membrane process. The flux decline can only be delayed with appropriate control and cannot be completely avoided [38]. 

Figure 10a also shows that M-15 has the highest permeability in the first filtration cycle, followed by M-0, M-5 and M-Ref. The high performance of M-15 is due to its large membrane pore size. However, after 180 min, M-5 shows the highest permeability towards the end of the filtration. According to Chollom et al. [39], the membrane with highest flux has a high tendency to suffer from more severe membrane fouling because of the great convective force which consequently brings foulants to the membrane surface. The finding demonstrates the advantage of high hydrophilicity posed by M-5 to allow it to maintain high permeability by water flushing as a simple operation for membrane fouling control. Overall, it can be conducted that the fabricated modified membranes show higher permeability as compared to the plain PVDF membrane (M-Ref), which remains as the lowest value of permeability throughout the filtration. 

Figure 10b exhibits the percentage of oil rejection for each membrane. Only three membranes achieve oil rejection of >80%. M-Ref has the lowest oil rejection, whereas M-5 provides the highest oil rejection. All VIPS membranes show better oil rejection than M-Ref. The separation of the oil/water emulsion is improved thanks to the presence of PVP in enhancing the hydrophilicity. This is because the residual PVP enhances the hydrophilicity that promotes the penetration of water through the membrane, whereas it repels the oil droplets, as also reported elsewhere [40]. The results of the particle size distribution of the oil droplets in the oil/water emulsion show that the sizes of the droplets are distributed in three modes with peaks around 0.25 (≈10%), 0.9 (≈45%) and 4.0 µm (≈45%). From the pore size perspective (Figure 4), those droplets would be fully rejected by M-Ref only, the largest group of droplets (4.0 µm) would be fully retained by M-0 and M-5 and the rests would have minimum retentions. Based on the results in Figure 10b, the high oil rejections can be explained by the hydrophilic membrane property that repels the droplets when approaching the membrane surface. Moreover, as reported by Huang et al. [40], the VIPS membrane has high membrane flux with low fouling due to the presence of hydrophilic functional groups in PVP as well as their higher pore size. Hence, this further justifies the following results where the modified membrane with prolonged exposure time can eventually lead to higher oil rejection along with high permeability.

#### 3.2.2. Fouling Resistance Analysis

**Figure 11:**
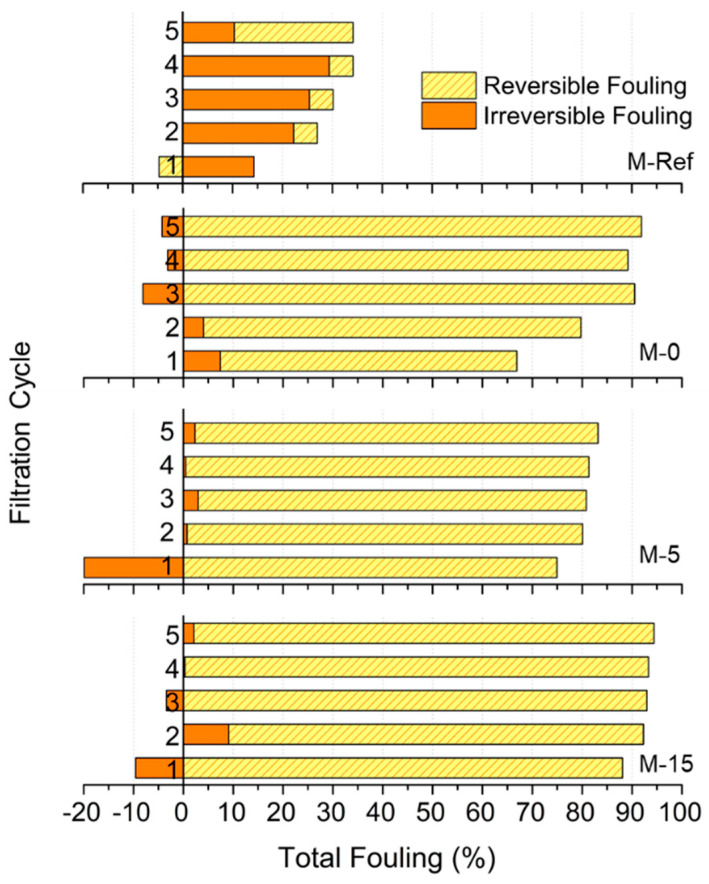
Fouling resistance analysis of the modified membranes.

Figure 11 depicts the effect of applying the VIPS method on the membrane antifouling properties. The antifouling properties were assessed based on their reversibility, in which reversible fouling can be eliminated by physical cleaning such as water flush, whereas irreversible fouling needs chemical cleaning [14]. However, the data of oil/water emulsion filtration suggests the advantage of M-5 over the other membranes in terms of permeability. In general, the higher total fouling shown by higher filtration cycles indicates the accumulation of fouling effects over the filtration cycles. 

From Figure 11, a clear contrast can be seen between the membrane with the lowest permeability (M-Ref) and the membrane with the highest permeability (M-5). Since M-Ref has the lowest permeability, it can be observed that it has the lowest fouling rate. However, the relatively low permeability obtained by the membrane made it less attractive for filtration. Meanwhile, when comparing M-0 and M-15, where the permeabilities of both membranes are almost the same, M-15 has higher total fouling as compared to M-0 after 5 cycles. The percentage of reversible fouling for M-0 is even higher than M-15, which is around 91.89% after five cycles. In this case, it is proven that the total fouling experience by M-0 is majorly influenced by reversible fouling. A study by Tsuyuhara et al. [41] suggests that permeability along with contact angle affect the degree of physically irreversible fouling, where this irreversible fouling might correspond to M-0 properties of relatively low surface contact angle (more hydrophilic) and greater clean water permeability. It explains why M-0 has higher reversible fouling as compared to M-15. 

## 4. Conclusions

This project demonstrates that the application of the VIPS fabrication method and the addition of PVP additives under extended exposure time dramatically change the membrane morphology from a typical porous morphology with macrovoids and a finger-like substructure, to one with a cellular structure. Consequently, the altered membrane properties also influence the membrane filterability as well as their antifouling properties. The results also suggest that the extension of exposure time beyond 30 min is inadvisable since it might deteriorate the membrane’s mechanical strength despite of its relatively large mean pore size. From this study, applying 5 min of exposure time before immersion (M-5) elevates the membrane’s clean water permeability by up to 84% compared to pristine PVDF membrane. When the exposure time is extended to 15 min (M-15), the clean water permeability is also enhanced thanks to its larger mean pore size. As expected, higher permeabilities were obtained by the membranes at the initial stage of oil/water emulsion filtration. However, this condition also unfortunately caused the membrane to suffer from more severe membrane fouling because of the great convective force, which consequently brings foulants to the membrane surface and leads to foulants’ accumulation. The best performing membrane was demonstrated by M-15, by showing oil/water emulsion permeability of 187 Lm^−2^ h^−1^ bar^−1^ and oil rejection of 91.3%. The performance was achieved thanks to combination of the structural properties (i.e., pore size) and hydrophilic property imposed by the residual PVP in the membarne matrix that limits the interaction of oil droplets with the membrane surface, and which lowers the membrane fouling and, at the same time, enhances oil rejection. Overall findings also demonstrate the ability of the VIPS method in tuning the morphological properties of the resulting membrane and improving their hydraulic performance. However, further studies on the membrane development for oil/water emulsion filtration are expected in the future in order to improve the feasibility of membrane technology for treating oily wastewater.

## Figures and Tables

**Table 1:** Summary of membrane composition and parameters.

Type of Membranes	Composition (wt.%)				Exposure Time before Immersion (minutes)
	PVDF *	PVP *	LiCl *	DMAC *	
M-Ref	15	0	0	85	0
M-0	13	3	0.1	83.9	0
M-5	13	3	0.1	83.9	5
M-15	13	3	0.1	83.9	15
M-30	13	3	0.1	83.9	30

* PVDF denotes as polyvinylidene fluoride, PVP as polyvinyl pyrrolidone, LiCl as lithium chloride and DMAC as dimethylacetamide.

**Table 2:** Elemental composition of the resulting membranes by energy dispersive X-ray (EDX) analysis.

Membrane	Composition (%)			
	Carbon (C)	Fluoride (F)	Oxygen (O)	Nitrogen (N)
M-Ref	55.55	42.84	1.61	0.00
M-0	56.04	41.11	2.85	0.00
M-5	52.93	45.36	1.71	0.00
M-15	56.11	38.81	4.29	0.79
M-30	60.66	33.61	4.73	1.00
